# Role of Sarcopenia in Advanced Malignant Cutaneous Melanoma Treated with Immunotherapy: A Meta-Analysis

**DOI:** 10.1159/000525928

**Published:** 2022-07-11

**Authors:** Alexey Surov, Hans-Jonas Meyer, Andreas Wienke

**Affiliations:** ^a^Department of Radiology and Nuclear Medicine, University of Magdeburg, Magdeburg, Germany; ^b^Department of Diagnostic and Interventional Radiology, University of Leipzig, Leipzig, Germany; ^c^Institute of Medical Epidemiology, Biostatistics, and Informatics, Martin-Luther-University Halle-Wittenberg, Halle (Saale), Germany

**Keywords:** Malignant melanoma, Sarcopenia, Survival, Toxicity

## Abstract

**Introduction:**

The role of sarcopenia in malignant cutaneous melanoma is unclear. The aim of the present meta-analysis was to analyze the prevalence and clinical role of sarcopenia in patients with advanced cutaneous melanoma based on a large cohort.

**Methods:**

MEDLINE, Cochrane, and SCOPUS databases were checked for relationships between sarcopenia and clinical outcomes in melanoma up to September 2021. Overall, 6 studies including 719 patients met the inclusion criteria. The meta-analysis was performed using RevMan 5.3 software.

**Results:**

The prevalence of sarcopenia was 40.23%. Sarcopenia did not influence dose-limiting toxicity of treatment, hazard ratio (HR) 1.01, 95% CI (0.70–1.47). Sarcopenia was associated with lower progression-free survival (PFS): HR 1.49, 95% CI (0.98–2.26), and lower overall survival (OS): HR 1.67, 95% CI (1.11–2.52).

**Conclusions:**

The cumulative prevalence of sarcopenia in malignant cutaneous melanoma is 40.77%. Sarcopenia is slightly associated with PFS and OS and it is not associated with treatment toxicity.

## Introduction

Sarcopenia plays an important role in oncology. There is great evidence based on large meta-analyses that sarcopenia can predict relevant outcomes in several tumors [1, 2, 3, 4, 5, 6]. For example, sarcopenia is associated with treatment toxicity in breast cancer and hepatocellular carcinoma [1, 2]. In patients who underwent surgical resection of tumors, sarcopenia predicts occurrence of major postoperative complications. So far, in esophageal cancer, sarcopenia is associated with overall morbidity (RR 1.16, 95% CI: 1.01–1.33), respiratory complications (RR = 1.64, 95% CI: 1.21–2.22), and anastomotic leaks (RR = 1.39, 95% CI: 1.10–1.76) [3]. Similar results were reported for gastric cancer and colorectal cancer [4, 5].

Furthermore, sarcopenia is a predictive factor for survival rates in oncology. Sarcopenia is associated with overall survival (OS) in several tumors. This phenomenon was observed in several tumors, for instance, in esophageal cancer: hazard ratio (HR) = 1.58; 95% CI: 1.35, 1.85 [3], gastric cancer: HR = 2.12, 95% CI: 1.89–2.38 [4], and pancreatic cancer: HR = 1.49; 95% CI: 1.27–1.74, *p* < 0.001 [6].

The current data about sarcopenia in malignant cutaneous melanoma are based on small studies and, therefore, cannot apply as evident. The purpose of the present meta-analysis was to provide evident data about associations between sarcopenia and clinical outcomes in patients with advanced cutaneous melanoma treated with immunotherapy.

## Materials and Methods

### Data Acquisition

**Fig. 1:**
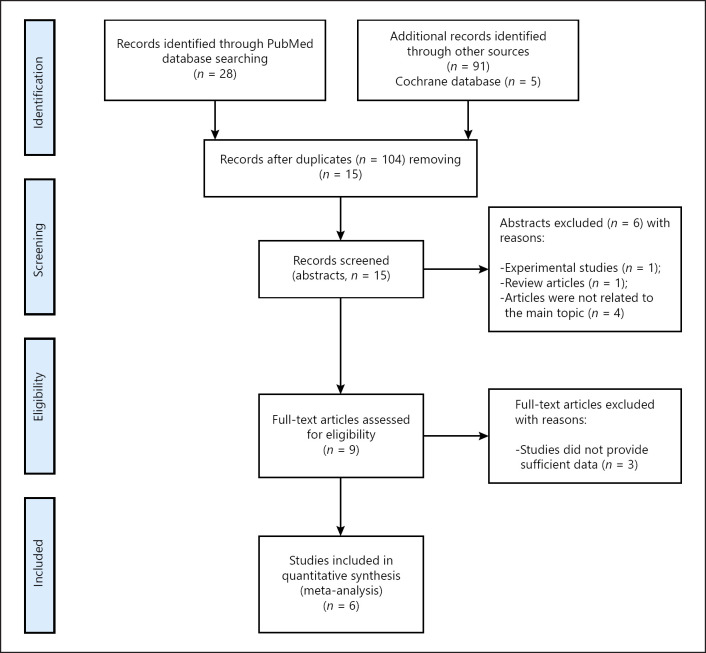
PRISMA flowchart of the data acquisition.

For the present analysis, a search in MEDLINE library, Cochrane, and SCOPUS databases was performed for studies analyzed associations between sarcopenia and relevant clinical outcomes in melanoma up to September 2021. All papers within the last 10 years were evaluated. Figure 1 demonstrates a flowchart of the data acquisition. For the data collection, the Preferred Reporting Items for Systematic Reviews and Meta-Analyses statement (PRISMA) was used [7]. The following search criteria were used: “sarcopenia OR low skeletal muscle mass OR body composition OR skeletal muscle index AND melanoma.”

The primary search identified 124 items.

Inclusion criteria for the present meta-analysis were as follows:

Original investigation involved human patients with cutaneous melanoma;
Estimation of pretreatment sarcopenia defined by CT images;
Treatment with immunotherapy in a palliative setting;
Reported data regarding influence of sarcopenia on relevant outcomes (hazard and/or odds ratios and 95% CI) including OS, progression-free survival (PFS), treatment toxicity;
Exclusion criteria were as follows:
Review articles and/or letters;
Case reports;
Non-English language;
Experimental studies;
Absence of statistical data about influence of sarcopenia on outcomes (hazard and/or odds ratios and 95% CI).

Duplicate articles (*n* = 109) were removed. Furthermore, the full texts of the remaining 15 articles were checked for possible data inclusion. Overall, 6 articles met the inclusion criteria [8, 9, 10, 11, 12, 13, 14]. The following data were acquired for the analysis: authors, year of publication, number of patients, prevalence of sarcopenia, and statistical data about influence of sarcopenia on relevant outcomes (hazard/odds ratios and 95% CI).

### Meta-Analysis

The methodological quality of the included 6 studies was checked by two observers (H.-J.M. and A.S.) using the Newcastle-Ottawa Scale (Table 2) [15]. The meta-analysis was performed by using the RevMan 5.3 software (Computer program, version 5.3. Copenhagen: The Nordic Cochrane Center, the Cochrane Collaboration, 2014) [16, 17]. Heterogeneity was calculated by means of the inconsistency index *I*^2^. Furthermore, DerSimonian and Laird [18] random-effects models with inverse-variance weights were performed without corrections.

## Results

### Included Studies and Patients

Overall, 6 studies were included into the present meta-analysis. All of them were retrospective. NOS values among the studies were low, indicating a low risk of bias (Table 1). The included studies comprised 719 patients. There were 271 women (37.7%) and 421 men (58.6%). In 27 patients (3.7%), the gender was not reported. The mean age of the patients was 60.4 ± 5.3 years. In all studies, sarcopenia was defined as low skeletal muscle mass on pretreatment staging computed tomography. Different tests for estimation of sarcopenia were performed (Table 2).

### Prevalence of Sarcopenia

**Fig. 2:**
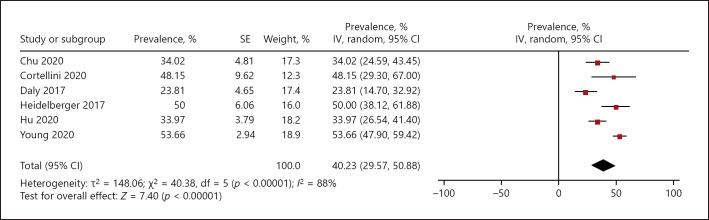
Reported prevalence of sarcopenia in patients with malignant cutaneous melanoma.

The prevalence of sarcopenia ranged from 23.81% to 53.66%. The cumulative calculated prevalence among the studies was 40.23%, 95% CI (29.57–50.88%) (Fig. 2).

### Treatment Toxicity

**Fig. 3:**
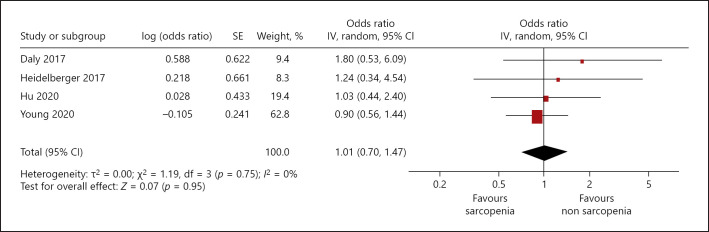
Association of sarcopenia with dose-limiting treatment toxicity in patients with malignant cutaneous melanoma, unadjusted HR.

Relationships between sarcopenia and occurrence of dose-limiting toxicity (DLT) were investigated in 4 studies (595 patients). In 3 studies, treatment with checkpoint inhibitors (nivolumab or pembrolizumab) and in one study, therapy with an anticytotoxic T-cell lymphocyte-4 monoclonal antibody (ipililumab) was performed. Sarcopenia was associated with DLT, HR 1.01, 95% CI (0.70–1.47) (Fig. 3). There was no heterogeneity between the studies (*I*^2^ = 0%).

### Progression Free Survival

**Fig. 4:**
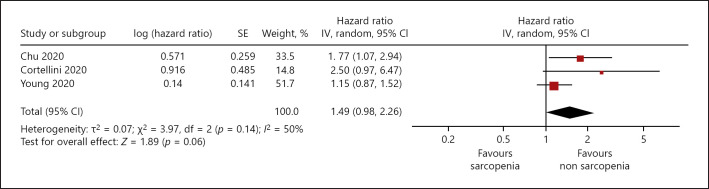
Association of sarcopenia with progression free survival in patients with malignant cutaneous melanoma, unadjusted HR.

Associations between PFS and sarcopenia were analyzed in 3 studies (simple regression) including 411 patients. Patients with sarcopenia showed lower PFS, HR 1.49, 95% CI (0.98–2.26) (Fig. 4). Heterogeneity between the studies was moderate (*I*^2^ = 50%).

### Overall Survival

**Fig. 5:**
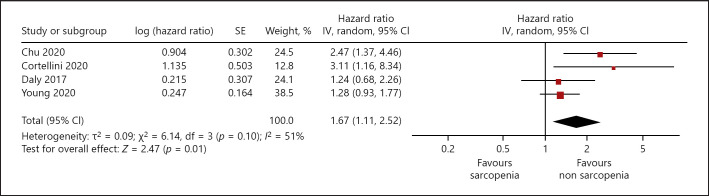
Association of sarcopenia with progression free survival in patients with malignant cutaneous melanoma, unadjusted HR.

In 4 studies (495 patients), influence of sarcopenia on OS was analyzed. Sarcopenia was associated with lower OS (simple regression), HR 1.67, 95% CI (1.11–2.52) (Fig. 5). Heterogeneity between the studies was moderate (*I*^2^ = 51%).

## Discussion

This is the first analysis regarding associations between sarcopenia identified on CT and relevant outcomes in malignant cutaneous melanoma treated with immunotherapy. As shown, sarcopenia is a frequent event in melanoma patient with an approximately prevalence of 40%. This value is comparable with the frequencies reported for other malignant tumors. For example, in malignant hematological diseases, it is 39.1% [19]. In esophageal cancer, it accounts 48% [20].

Furthermore, the present analysis shows that LSMM is only slightly associated with relevant outcomes in melanoma patients treated with immunotherapy. Notably, in contrast to other malignancies, the identified heterogeneity among the studies is low or moderate according to the guidelines from the Cochrane handbook [21]. This suggests that the present results can be applied as true. Interestingly, the identified associations between LSMM and clinical outcomes in melanoma are lower than those in other frequent malignant tumors. So far, in lung cancer, sarcopenia is associated with a shorter OS, HR, 2.23; 95% CI: 1.68–2.94 [22]. Similar results were reported for gastric cancer, colorectal cancer, and pancreatic cancer [4, 5, 6]. This finding indicates that LSMM represents no great limitation in advanced malignant melanoma.

According to the literature, LSMM influences significantly DLT on chemotherapy [1]. For example, in breast cancer patients, sarcopenic patients had more grade 3–5 toxicity compared to nonsarcopenic patients with a risk ratio of 2.17, 95% CI (1.4–3.34) [1]. In metastatic colorectal cancer treated with regorafenib, LSMM showed a stronger association with DLT: OR = 15.60, 95% CI (1.72–141.17) [23]. Interestingly, the influence of sarcopenia on treatment related toxicity in melanoma is very low. It may be related to the fact that in the present analysis, relationships between treatment toxicity and LSMM were analyzed in patients received immunotherapy. This finding is in agreement with a recent large meta-analysis that showed that LSMM predicted DLT in patients treated with conventional (5-fluoruracil and/or platin-based) chemotherapy and several kinase inhibitors but not in patients treated with immunotherapy [24].

The results of the present analysis indicate radiological reports should also provide information regarding body composition. The estimation of skeletal muscle mass is not complex and is a by-product of staging computer tomography.

Importantly, LSMM/sarcopenia is a potential modifiable factor [25, 26]. Some previous studies showed that exercise and nutritional support program can reduce sarcopenia and improve clinical outcomes in oncologic patients [25, 26].

There are some limitations of the present analysis to address. All included studies are retrospective. Furthermore, the analysis includes only studies in English language. Additionally, some studies have patient selection bias. Finally, different measurements and cut-offs for LSMM/sarcopenia were used in the studies. However, this meta-analysis is based on the largest cohort to date and provides evident data about the role of LSMM in melanoma. Clearly, large prospective studies are needed to confirm our results.

In conclusion, the cumulative prevalence of LSMM in malignant cutaneous melanoma is 40.23%. LSMM is slightly associated with PFS and OS and is not associated with treatment toxicity.

## Statement of Ethics

An ethics statement is not applicable because this study is based exclusively on published literature.

## Conflict of Interest Statement

The authors have no conflict of interest to declare.

## Funding Sources

No funding was received.

## Author Contributions

Alexey Surov: study concept and design, acquisition of data, statistical analysis, and drafting of the manuscript. Hans-Jonas Meyer: acquisition of data and critical revision. Andreas Wienke: acquisition of data and statistical analysis.

## Data Availability Statement

All data generated or analyzed during this study are included in this article. Further inquiries can be directed to the corresponding author.

## Figures and Tables

**Table 1:** The quality of the studies by NOS scale

Study	Representativeness of the exposed cohort	Selection of the nonex-posed cohort	Ascertainment of exposure	Demonstration that outcome of interest was not present at start of study	Comparability of cohorts on the basis of the design or analysis	Assessment of outcome	Was follow-up long enough for outcomes to occur	Adequacy of follow-up of cohorts	Quality score
Chu et al. [8], 2020	*	*	*	*	**	*	*	*	9
Cortellini et al. [9], 2020	*	*	*	*	**	*	*	*	9
Daly et al. [10], 2017	*	*	*	*	**	*	*	*	9
Heidelberger et al. [11],	*	*	*	*	*		*	*	7
2017									
Hu et al. [12], 2020		*	*	*	*	*	*	*	7
Young et al. [14], 2020	*	*	*	*	**	*	*	*	9

NOS, Newcastle-Ottawa Scale.

**Table 2:** Data regarding involved studies

Authors, year	Design	Patients	Patients with sarcopenia, *n* (%)	Test for sarcopenia and threshold values	Treatment	Analyzed clinical values
Chu et al. [8], 2020	Retrospective	97	33 (34.02)	SMD (density), no threshold values provided	Iplimumab	Prevalence, OS, PFD

Cortellini et al. [9]	Retrospective	27	13 (48.1)	SMI; overweight (BMI ≥ 25): male, 50.2 cm^2^/m^2^; female, 59.6 cm^2^/m^2^; non-overweight (BMI < 25): male, 48.4 cm^2^/m^2^; female, 36.9 cm^2^/m^2^	PD 1/PD L1 inhibitors	Prevalence, OS, PFS*

Daly et al. [10] 2017	Retrospective	84	20 (21.3)	Mean muscle area, gender- and BMI-specific cut-points were used to define sarcopenia and low muscle area	Ipilimumab	Prevalence, OS, toxicity

Heidelberger et al. [11], 2017	Retrospective	68	43 (62.2)	SMI, 52.4 cm^2^/m^2^ for men and 38.5 cm^2^/m^2^ for women	Nivolumab or pembrolizumab	Prevalence

Hu et al. [12], 2020	Retrospective	156	53 (34)	PMI, lower third was considered sarcopenic	Pembrolizumab	Prevalence, OS, PFS, toxicity

Young et al. [14], 2020	Retrospective	287	133 (46.3)	SMI, for patients with BMI <25, sarcopenia was defined as SMI <43 cm^2^/m^2^ for men and <41 cm^2^/m^2^ for women and for BMI ≥26, sarcopenia was defined as <53 cm^2^/m^2^ for men and <41 cm^2^/m^2^ for women	Ipilimumab + nivolumab Pembrolizumab Nivolumab Atezolizumab	Prevalence, OS, PFS

BMI, body mass index; SMD, skeletal muscle density; PMI, psoas muscle index; SMI, skeletal muscle index; DFS, disease free survival; OS, overall survival; DLT, dose-limiting toxicity.

* Data provided by the authors by request.
